# PSRR: A Web Server for Predicting the Regulation of miRNAs Expression by Small Molecules

**DOI:** 10.3389/fmolb.2022.817294

**Published:** 2022-03-21

**Authors:** Fanrong Yu, Bihui Li, Jianfeng Sun, Jing Qi, Rudy Leon De Wilde, Luz Angela Torres-de la Roche, Cheng Li, Sajjad Ahmad, Wenjie Shi, Xiqing Li, Zihao Chen

**Affiliations:** ^1^ Department of Obstetrics and Gynecology, Fengxian District Central Hospital, Shanghai Jiao Tong University Affiliated to Sixth People’s Hospital South Campus, Shanghai, China; ^2^ Department of Oncology, The Second Affiliated Hospital of Guilin Medical University, Guilin, China; ^3^ Department of Bioinformatics, Wissenschaftzentrum Weihenstephan, Technical University of Munich, Freising, Germany; ^4^ Institute for Transplantation Diagnostics and Cell Therapeutics, Medical Faculty, Heinrich Heine University Düsseldorf, Moorenstraße, Düsseldorf, Germany; ^5^ University Hospital for Gynecology, Pius-Hospital, University Medicine Oldenburg, Oldenburg, Germany; ^6^ Department of Orthopaedic Surgery, Beijing Jishuitan Hospital, Fourth Clinical College of Peking University, Beijing, China; ^7^ Department of Health and Biological Sciences, Abasyn University, Peshawar, Pakistan; ^8^ Oncology Department, Henan Provincial People’s Hospital, Zhengzhou University People’s Hospital, Zhengzhou, China

**Keywords:** microRNA, small molecule, machine learning, web server, endometrial cancer

## Abstract

**Background:** MicroRNAs (miRNAs) play key roles in a variety of pathological processes by interacting with their specific target mRNAs for translation repression and may function as oncogenes (oncomiRs) or tumor suppressors (TSmiRs). Therefore, a web server that could predict the regulation relations between miRNAs and small molecules is expected to achieve implications for identifying potential therapeutic targets for anti-tumor drug development.

**Methods:** Upon obtaining positive/known small molecule-miRNA regulation pairs from SM2miR, we generated a multitude of high-quality negative/unknown pairs by leveraging similarities between the small molecule structures. Using the pool of the positive and negative pairs, we created the Dataset1 and Dataset2 datasets specific to up-regulation and down-regulation pairs, respectively. Manifold machine learning algorithms were then employed to construct models of predicting up-regulation and down-regulation pairs on the training portion of pairs in Dataset1 and Dataset2, respectively. Prediction abilities of the resulting models were further examined by discovering potential small molecules to regulate oncogenic miRNAs identified from miRNA sequencing data of endometrial carcinoma samples.

**Results:** The random forest algorithm outperformed four machine-learning algorithms by achieving the highest AUC values of 0.911 for the up-regulation model and 0.896 for the down-regulation model on the testing datasets. Moreover, the down-regulation and up-regulation models yielded the accuracy values of 0.91 and 0.90 on independent validation pairs, respectively. In a case study, our model showed highly-reliable results by confirming all top 10 predicted regulation pairs as experimentally validated pairs. Finally, our predicted binding affinities of oncogenic miRNAs and small molecules bore a close resemblance to the lowest binding energy profiles using molecular docking. Predictions of the final model are freely accessible through the PSRR web server at https://rnadrug.shinyapps.io/PSRR/.

**Conclusion:** Our study provides a novel web server that could effectively predict the regulation of miRNAs expression by small molecules.

## Introduction

MicroRNAs (miRNAs) are a novel class of non-coding RNAs of ∼ 22 nucleotides (He et al., 2004). A silencing complex formed by miRNA and the Argonaute (AGO) protein could reduce gene expression by repressing translation and accelerating mRNA degradation (Jonas and Izaurralde, 2015). miRNAs play crucial roles in multiple biological processes, such as cell apoptosis, proliferation, and differentiation because of the post-transcriptional regulatory mechanisms (He et al., 2004). Each miRNA could control more than 200 mRNAs (Yamada et al., 2019), and more than one-third of protein-encoding genes could be down-regulated by miRNAs (Bartel et al., 2009). The first miRNA, lin-4, was discovered in the early 1990s (Lee et al., 1993), and studies related to miRNAs have grown exponentially in recent years (Gammell, 2007). Many studies disclosed that dysregulation of miRNAs is implicated in various diseases (Liu et al., 2018), imparting them with a role of being potential therapeutic targets (Guan and Disney, 2012).

RNA forms a complex tertiary structure with specific deep pockets that enable small molecules to bind with high selectivity. For example, the clinical use of Linezolid, a small molecule antibiotic targeting RNA, suggested that RNAs are important drug targets like proteins (Warner et al., 2018). Recently, an approach termed “Inforna” was developed to design small molecules targeting RNAs by sole sequences (Velagapudi et al., 2014). Inforna also successfully developed a small molecule that could bind tightly and specifically to microRNA-96, which verified the reliability and feasibility of computational-base design or selection of small molecules binding to miRNAs (Velagapudi et al., 2016). Other studies provided computational models for predicting interactions between miRNAs and small molecules (Qu et al., 2018; Jamali et al., 2020; Deepthi and Jereesh, 2021). However, there are three major disadvantages of these models. First, these models could only predict the binding of small molecules with miRNAs but fail to predict the regulation of miRNA expression (up-regulation/down-regulation) by small molecules. The regulation role is crucial for anti-tumor drug development because miRNAs may function as oncogenes (oncomiRs) or tumor suppressors (TSmiRs). Second, it is difficult for others to use these models since online web servers are not available for these models. Besides, these models contain complex processing procedures such as miRNA structure prediction and energy minimum (Disney et al., 2016). Thus, a user-friendly and high-accuracy web server that allows designing small molecules increasing or decreasing miRNA expression is urgently needed.

Here, we established a powerful and user-friendly web server for predicting the regulation of miRNA expression by small molecules. 4,132 up-regulation pairs (2066 positive/known pairs and 2066 negative/unknown pairs) and 3,182 down-regulation pairs (1,591 positive/known pairs and 1,591 negative/unknown pairs) were used for constructing an up-regulation model and a down-regulation model, respectively. Among five advanced machine learning algorithms, the random forest algorithm demonstrating the best performance was introduced to build the up-regulation and down-regulation prediction models. Predictions from the web server were finally generated based on the random forest algorithm. Furthermore, we expanded the dataset for the web server to 1509 FDA approved small molecules and 2,236 human miRNAs to provide more drug candidates and targets.

## Material and Methods

### The Downloading of Known/Positive Regulation Pairs of Small Molecules and miRNAs

In the current study, the known SM-miRNA regulation pairs were downloaded from the SM2miR database (Liu et al., 2013). Up-regulation pairs were separate from down-regulation pairs, which were referred to as Dataset1 and Dataset2, respectively. The SM-miRNA pairs with missing miRNA sequences in Dataset1 and Dataset2 were deleted, which finally left 1,102 miRNAs, 170 small molecules and 2066 up-regulation pairs for Dataset1, and 869 miRNAs, 150 small molecules and 1,591 down-regulation pairs for Dataset2. All these known pairs were labeled as positive SM-miRNA pairs.

### The Generation of Unknown/Negative SM-miRNA Regulation Pairs

Currently, negative/unknown SM-miRNA regulation pairs which were necessary for prediction model construction were not available. Similar to a previous study that leveraged molecular similarities to build highly credible negative compound–protein interactions (Liu et al., 2015), we constructed a regulation scoring approach (described below in detail) based on similarity scores between small molecules to screen negative SM-miRNA regulation pairs by estimating a regulation score of these miRNAs. The regulation scoring approach was made upon the assumption that small molecules with similar chemical structures are more likely to regulate the same miRNAs. Thus, a small molecule is unlikely to increase the specific miRNA expression if this small molecule is found to have a significantly different chemical structure to the small molecule promoters of the miRNA. The specific procedures of screening negative samples are listed below.

We used the “CDK” R package to compute a 166-bit MACCS fingerprint vector (Guha, 2016) based on the simplified molecular-input line-entry system (SMILES) string of each small molecule. To measure the similarity of a pair of small molecules, we computed the Tanimoto coefficient that is one way to quantify their similarity based on their respective fingerprints (Bajusz et al., 2015). Then, the regulation scores between any small molecules and any miRNAs were calculated. For example, we assumed that the relationship (say regulation profiles) between a small molecule D (smD) and miRNA001 was unknown, but small molecules A (smA), B (smB), and C (smC) were known to increase the expression of miRNA001. The regulation score $r_{D}^{\text{miRNA}001}$ of the miRNA001 expression increased by smD is computed by

$$r_{D}^{\mathrm{miRNA}001}\underset{=}{def}\frac{{\bar{s}}_{A}+{\bar{s}}_{B}+{\bar{s}}_{C}}{3}=\bar{s}$$

where $\bar{s}$ represents the mean value of Tanimoto coefficients of smA-smD, smB-smD, and smC-smD. Note that we illuminated that the regulation scoring approach is a trade-off scheme in the absence of mature methods available for this purpose. Similarly, we repeated this procedure for the rest of miRNAs. The pairs with regulation score less than 0.1 were defined as potential negative regulation pairs. It has been well established that a comparable ratio between positive and negative samples is conducive to training a prediction model. Therefore, among these potential negative regulation pairs, 2066 and 1,591 pairs were randomly selected for Dataset1 and Dataset2, respectively. Thus, Dataset1 included 2066 positive pairs and 2066 negative pairs. Dataset2 included 1,591 positive pairs and 1,591 negative pairs.

### Calculation of miRNA Descriptors and Small Molecules Descriptors

The sequences of miRNAs were downloaded from the miRbase (15 March 2021) database which encompasses the most complete miRNA information, containing miRNA sequences of 38,589 hairpin precursors and 48,860 mature microRNAs from 271 organisms (Kozomara et al., 2019). miRbase is accessible free of charge *via* the web server http://www.mirbase.org/. Firstly, we pulled from miRbase the information of miRNA sequences including miRNA sequence length. Secondly, we calculated the ratios of A, C, G and U as well as the frequencies of 2-mer miRNA patterns including AA, AC, … UC, and UU. Then, we extracted the frequencies of 4-mer miRNA patterns including AAAA, AAAC, … UUUC, and UUUU. The descriptor of sole miRNA sequence was finally represented by a 277-dimensional vector. The small molecule descriptors were obtained from the computed 166-dimensional MACCS fingerprint vector.

### Model Construction

We separately trained classifiers for predicting the up-regulation pairs in Dataset1 and down-regulation pairs in Dataset2. Dataset1 and Dataset2 were evenly divided into the training dataset (50%) and the testing dataset (50%). Machine learning classifiers used in our research were constructed and evaluated using a fully-automated algorithm-oriented R package, termed caret (Kuhn, 2008). Five-fold cross-validation (CV) was applied to determine model parameters based on our training dataset. Machine learning methods including Generalized Linear Models (GLM), K-nearest Neighbors (KNN), Support Vector Machine (SVM), Artificial Neural Network (ANN), and Random Forest (RF) were selected for model construction. The parameters were all tuned and optimized based on caret*.* For GLM, we optimized the parameter “alpha”. For KNN, the optimized parameter was “K value”. For SVM, we optimized the regularization parameter “C”. For ANN, the parameter “decay” was optimized. For RF, we adjusted regularization parameter “mtry”. The rest of all parameters of these classifiers are employed by default. The machine learning classifiers were trained by the parameter which makes the machine learning method reach the highest Area under the Curve (AUC) value calculated by five-fold CV in the training dataset. Then, we estimated the performance of classifiers in testing dataset by AUC value in the testing dataset. The up-regulation prediction classifier and the down-regulation prediction classifier with the highest AUC values in the testing dataset were selected for web server development.

### Web Server Development

To facilitate rapid prediction of the SM-miRNA by our models, we have developed and deployed a free, publicly available web-based Shiny application called Prediction of SM-miRNA Regulation pairs (PSRR) for providing and visualizing the results of our analyses. There is no login requirement for accessing any features in PSRR. It has been tested rigorously and extensively in different computer systems and popular web browsers such as Chrome, Firefox, and Internet Explorer. The PSRR output consists of tables and figures. The tables are generated by the “DT” R package that allows data querying and selection.

### Validation of Prediction Models on Independent Dataset

The prediction accuracy of up-regulation and down-regulation models was investigated on data collated from a review that summarized the developed small molecules to regulate the miRNAs expression (Van Meter et al., 2020). The sequence of miRNAs and the SMILES strings of small molecules were taken as input into our PSRR that output predicted rates.

### Data Acquisition and Global Gene Expression Analysis

The level-3 RNA-sequencing data was obtained from The Cancer Genome Atlas (TCGA). The “edgeR” R package was selected for miRNA differential expression analysis (Robinson et al., 2010). The inclusion criterion for selecting differentially expressed miRNAs (DEmiRNAs) was based on *p*-value < 0.05 and |log2FoldChange| > 1. Overall survival (OS) data of Uterine Corpus Endometrial Carcinoma (UCEC) patients were also available from TCGA. For each miRNA, the association between miRNAs and OS was determined using univariate cox regression. In tumor samples, the up-regulated miRNAs demonstrating *p*-value < 0.05 and hazards ratio (HR) > 1 in univariate cox regression analysis were defined as oncogenic miRNAs (oncomiRs). The Venn diagram was also introduced.

### Preparation of Structure and Molecular Docking

To validate the binding of predicted small molecules with oncomiRs obtained from the TCGA-UCEC study, molecular docking was performed in two steps as follows: 1) The 3D structures of miRNAs were predicted by a freely available server “RNAComposer” (Biesiada et al., 2016) and the structures of small molecules were downloaded from the PubChem database (Wang et al., 2009); 2) Molecular docking analysis was performed by AutoDock (Rizvi et al., 2013). The binding sites with the lowest binding energies were plotted by Pymol (Seeliger and de Groot, 2010).

## Results

### Preprocessing of the Source Data

**FIGURE 1:**
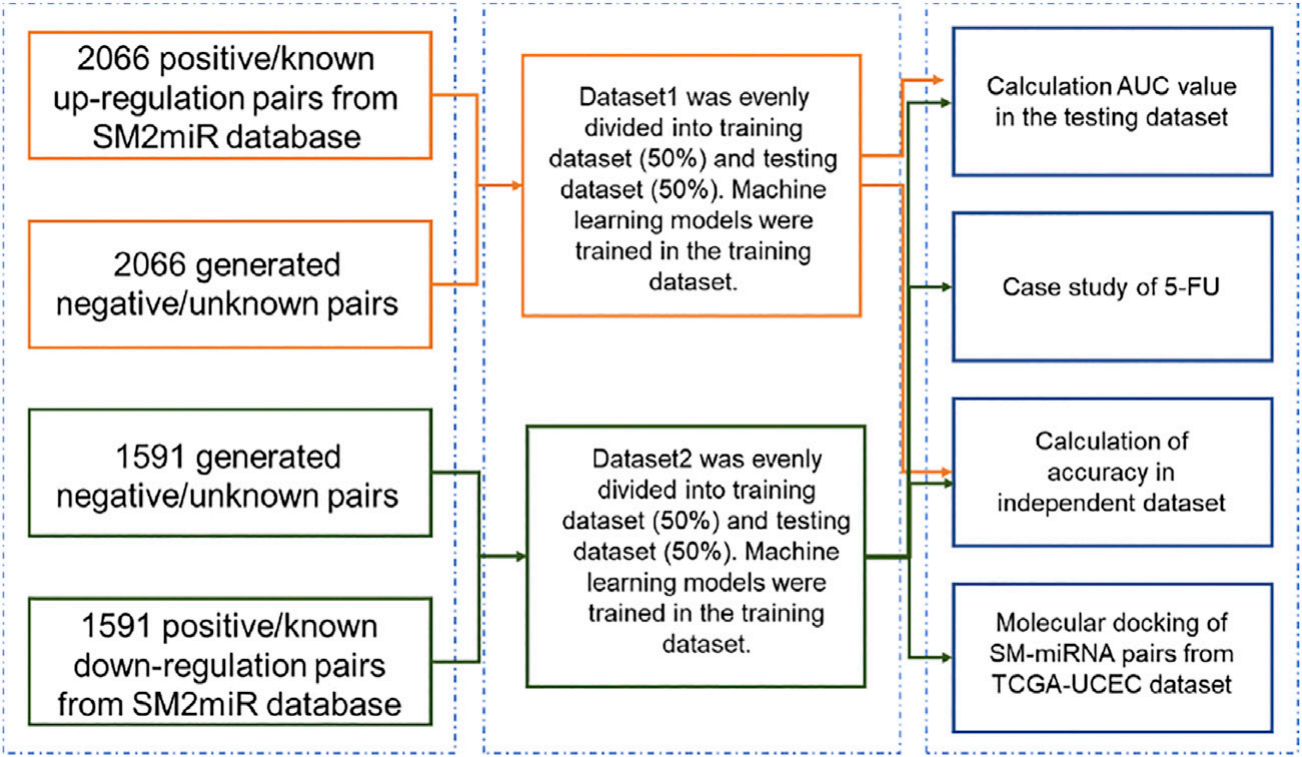
Flowchart of developing models for SM-miRNA regulation prediction.

**FIGURE 2:**
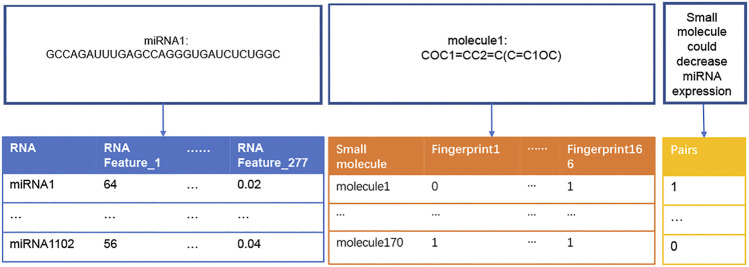
Construction of descriptors of miRNAs and small molecules. The table in blue presents a vector of 277 RNA features (i.e*.*, descriptors) for each miRNA sequence. For example, RNA Feature_1 represents the length of a miRNA sequence, and RNA Festure_277 represents the frequency of ‘UUUU’ in the miRNA sequence. The table in orange presents a vector of 166 fingerprints transformed from the SMILES string of each small molecule. “0” and “1” represent the absence and presence of a substructure in each small molecule, respectively. The table in yellow presents the ‘0’ and “1” labels for negative (not binding) and positive (binding) regulation pairs of miRNAs and small molecules, respectively.

The flowchart of this study was illustrated in Figure 1. Dataset1 (2096 positive pairs and 2096 artificially constructed negative pairs) was used to construct models to predict the up-regulation pairs of small molecule and miRNAs. Similarly, Dataset2 (1,591 positive and 1,591 negative pairs) was used to construct models to predict down-regulation pairs. As shown in Figure 2, we calculated the miRNA descriptors characterized by a 277-dimensional vector for each miRNA including miRNA sequence length, the ratios of A, C, G and U, the frequency of 2-mer miRNA patterns (AA, AC, … UC, and UU) and 4-mer miRNA patterns (AAAA, AAAC, … UUUC, and UUUU).

The descriptor of each small molecule was originated from SMILES data. SMILES, proposed by Weininger (David, 1988), could represent all atoms from a small molecule. The SMILES string of each small molecule was transformed into a MACCS molecular fingerprint that described the presence or absence of a substructure in this small molecule (Rogers and Hahn, 2010). In this study, 166-bit MACCS fingerprint vectors were selected as the descriptors for each small molecule (Figure 2).

### Construction and Validation of Machine Learning Models

**FIGURE 3:**
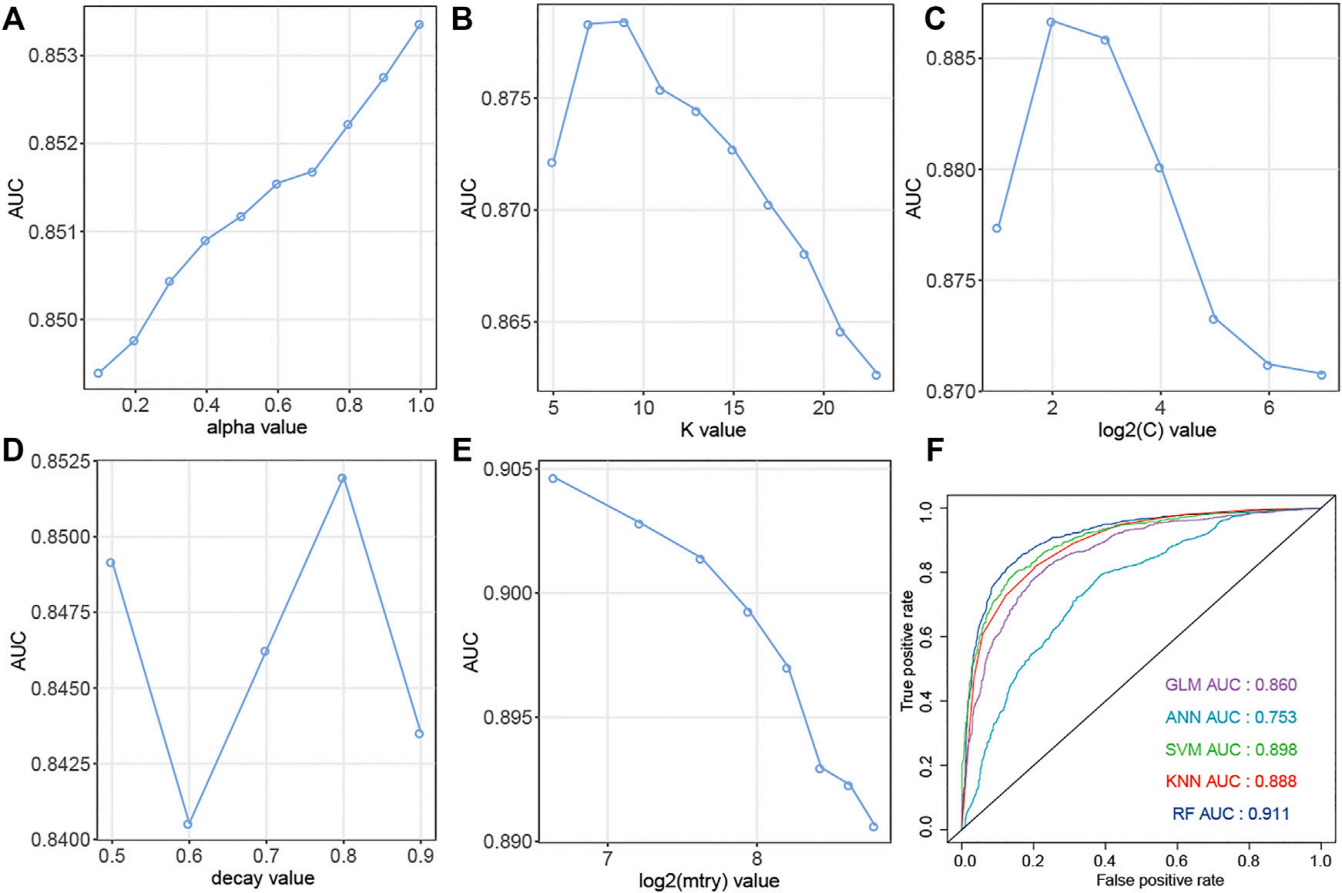
Prediction performance of models for SM-miRNA up-regulation pairs. **(A**–**E)** AUC values of 5 machine learning algorithms are generated by the 5-fold cross validation on the training dataset. **(A)** Generalized Linear Models (GLM). **(B)** K-nearest Neighbors (KNN). **(C)** Support Vector Machine (SVM). **(D)** Gradient Boosting Machine (GBM). **(E)** Random Forest (RF). **(F)** AUC values of 5 models were calculated on the testing dataset.

The up-regulation pairs from Dataset1 were randomly split into training (50%) and the testing sets (50%), respectively. We selected five popular machine learning methods including Generalized Linear Models (GLM), K-nearest Neighbors (KNN), Support Vector Machine (SVM), Artificial Neural Network (ANN), and Random Forest (RF) to initial construction of our models. The classifiers were firstly trained on the training set using a 5-fold cross-validation procedure to select the optimal parameters based on AUC values. In the model training phase, as shown in Figures 3A–E, the highest AUC values for GLM, KNN, SVM, ANN, and RF were 0.853, 0.878, 0.887, 0.852, 0.905 when the parameters “alpha”, “K value”, “log2(C)”, “decay” and “mtry” for GLM, KNN, SVM, ANN, and RF were set as “1”, “7”, “2”, “0.8” and “100”, respectively. In addition to using the AUC criterion, we also monitored sensitivity (Sens), specificity (Spec), AUC standard deviation (AUCSD), sensitivity standard deviation (SensSD) and specificity standard deviation (SpecSD) of models from 5-fold cross-validation in the training dataset as shown in the Table 1. Among these five machine learning classifiers, RF was selected as our representative model since it reached the highest AUC value at 0.905 when it was trained with “mtry” of “100” by 5-fold cross validation (Figure 3E). In the testing phase, the AUC values for GLM, KNN, SVM, ANN, and RF on the testing dataset were 0.860, 0.888, 0.898, 0.753 and 0.911 (Figure 3F), respectively. Thus, RF was the most accurate model for predicting up-regulation pairs because of both evidence of its highest AUC values in model training (0.905) and testing phases (0.911).

**TABLE 1:** Prediction performance of models for up-regulation pairs using the 5-fold cross validation on the training dataset.

Model	ROC	Sens	Spec	ROCSD	SensSD	SpecSD
GLM	0.853	0.770	0.767	0.0247	0.0263	0.0447
KNN	0.878	0.783	0.804	0.0151	0.0338	0.0403
SVM	0.887	0.812	0.814	0.0232	0.035	0.0506
ANN	0.852	0.798	0.727	0.0205	0.0439	0.0486
RF	0.905	0.825	0.823	0.00718	0.0131	0.0178

**FIGURE 4:**
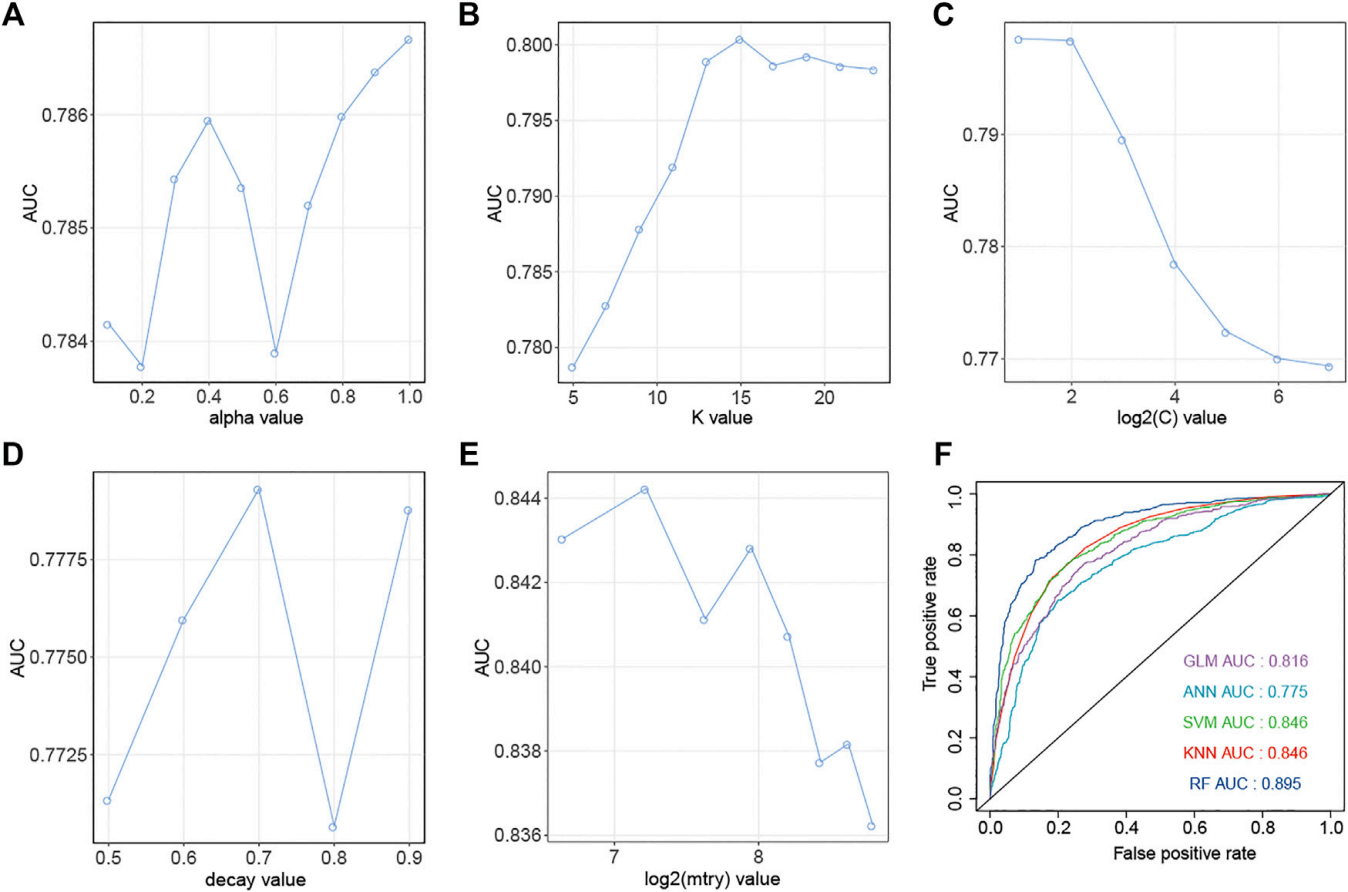
Prediction performance of models for SM-miRNA down-regulation pairs. **(A–E)** AUC values of 5 machine learning algorithms are generated by the 5-fold cross validation on the training dataset. **(A)** Generalized Linear Models (GLM). **(B)** K-nearest Neighbors (KNN). **(C)** Support Vector Machine (SVM). **(D)** Gradient Boosting Machine (GBM). **(E)** Random Forest (RF). **(F)** AUC values of 5 models were calculated on the testing dataset.

Similarly, down-regulation pairs from Dataset2 were also randomly split into training (50%) and testing sets (50%). The highest AUC values for GLM, KNN, SVM, ANN, and RF were 0.787, 0.800, 0.799, 0.771 and 0.844 (Figures 4A–E) when the parameters “alpha”, “K value”, “log2(C)”, “decay” and “mtry” for GLM, KNN, SVM, ANN, and RF were set as “1”, “15”, “2”, “0.5” and “149”, respectively. AUC, Sens, Spec, AUCSD, SensSD and SpecSD of models were shown in Table 2. Setting the “mtry” parameter to “100” made RF the best model reaching the highest AUC value at 0.905 among these five machine learning classifiers by 5-fold cross-validation (Figure 3E). In the testing phase, the AUC values for GLM, KNN, SVM, ANN, and RF were 0.816, 0.846, 0.846, 0.775 and 0.895 (Figure 4F), respectively. Thus, the most accurate RF model was finally chosen for predicting down-regulation pairs.

**TABLE 2:** Prediction performance of models for down-regulation pairs using the 5-fold cross validation on the training dataset.

Model	ROC	Sens	Spec	ROCSD	SensSD	SpecSD
GLM	0.787	0.684	0.776	0.028	0.059	0.0302
KNN	0.800	0.646	0.787	0.0149	0.0275	0.0335
SVM	0.799	0.672	0.777	0.0144	0.0349	0.0205
ANN	0.779	0.701	0.719	0.0434	0.0861	0.0365
RF	0.844	0.742	0.803	0.024	0.0411	0.052

Moreover, we calculated F1 scores based on different threshold values (Supplementary Table S1). The down-regulation prediction model reaches the maximum F1 score of 0.8176991 when the threshold is set to 0.41. The up-regulation prediction model reaches the maximum F1 score of 0.8468809 when the threshold is set to 0.48. Thus, the suggestion rate for down-regulation model is 0.41, and the suggestion rate for up-regulation model is 0.48. If the rate of the SM-miRNA pair predicted by our model is higher than the suggestion rate, this SM-miRNA regulation pair is worth studying.

### Case Study and Input Requirement of PSRR

CID3385 (5-FU) is one of the widely used chemotherapeutic drugs in the treatment of cancers. It could inhibit tumor growth by the reduction of DNA synthesis and the promotion of DNA damage (Bash-Imam et al., 2017). Based on the constructed down-regulation pair prediction model, we generated potential miRNAs that were predicted to be decreased by 5-FU. The top 10 miRNAs with the highest rates predicted by our model were found to be decreased by 5-FU (Table 3).

**TABLE 3:** Top 10 predicted miRNAs with highest probabilities are found to be experimentally decreased by CID3385.

Drug	miRNA	Possibility	Prediction result	References
CID3385	hsa-miR-27b-3p	0.990	Positive	Rastogi et al. (2014)
CID3385	hsa-miR-27a-3p	0.988	Positive	Shah et al. (2011)
CID3385	hsa-let-7e-5p	0.962	Positive	Shah et al. (2011)
CID3385	hsa-miR-15b-5p	0.958	Positive	Shah et al. (2011)
CID3385	hsa-miR-125a-5p	0.944	Positive	Shah et al. (2011)
CID3385	hsa-miR-200b-3p	0.944	Positive	Rossi et al. (2007)
CID3385	hsa-let-7a-5p	0.942	Positive	Shah et al. (2011)
CID3385	hsa-miR-374b-5p	0.94	Positive	Shah et al. (2011)
CID3385	hsa-miR-224–5p	0.936	Positive	Rossi et al. (2007)
CID3385	hsa-miR-21–5p	0.934	Positive	Rossi et al. (2007)

**FIGURE 5:**
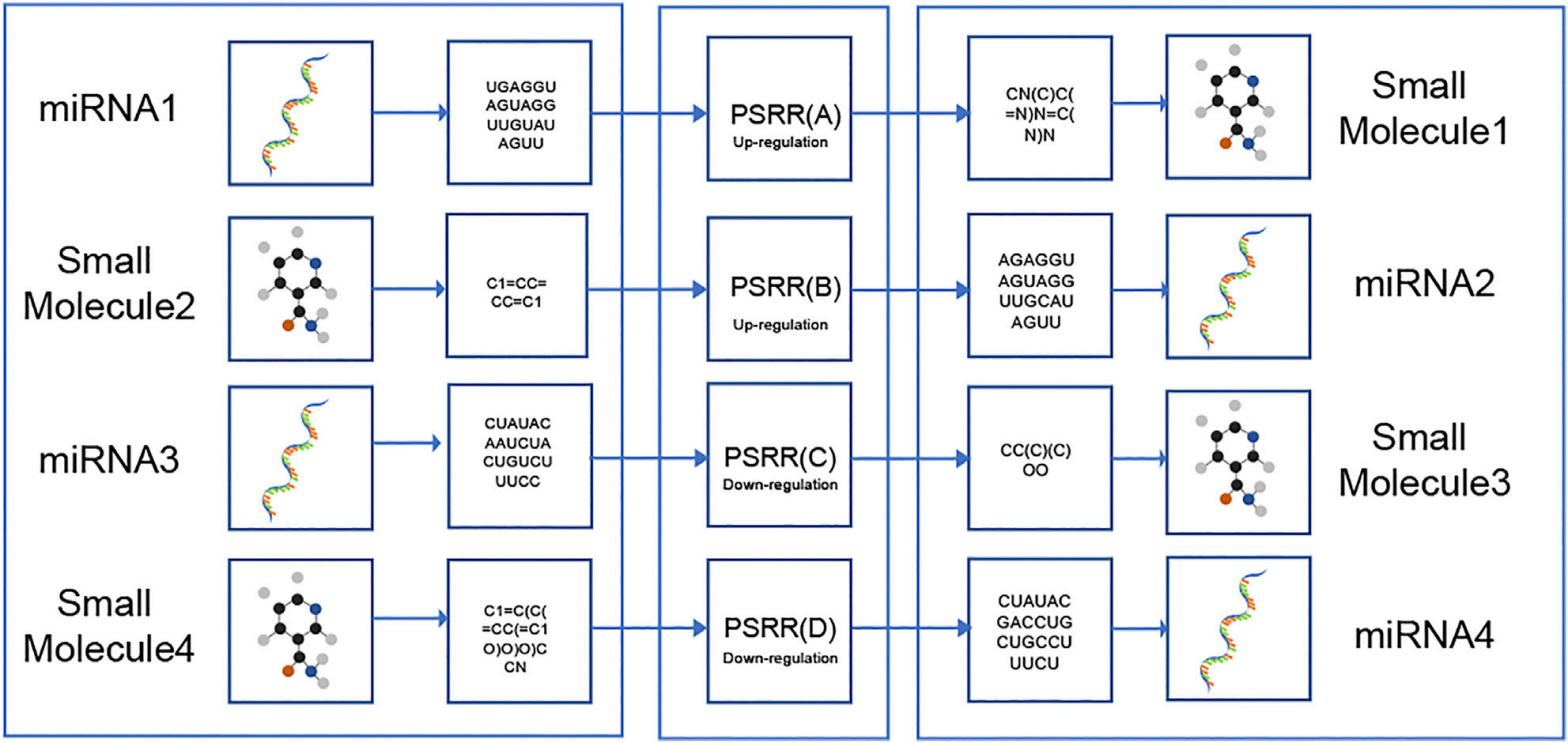
Workflow of PSRR. The PSRR **(A)** module takes as input a miRNA sequence and yields SMILES strings of small molecules that could increase the expression of this miRNA. The PSRR **(B)** module takes as input the SMILES string of a small molecule and yields sequences of miRNAs that could be decreased by this small molecule. The PSRR **(C)** module takes as input a miRNA sequence and yields SMILES strings of small molecules that could decrease the expression of this miRNA. The PSRR **(D)** module takes as input the SMILE string of a small molecule and yields sequences of miRNAs that could be decreased by this small molecule. In all the four modules, predicted regulation probabilities (0–1) were generated and given.

Using the “shiny” package in R language, a web server (PSRR, see *Web Server Development* Section) that contains the constructed model to predict the SM-miRNAs regulation pairs was built. The web server can be accessed *via* https://rnadrug.shinyapps.io/PSRR/. Alternatively, either sequences of miRNAs or SMILES strings of small molecules are necessarily needed as input to the web server, with 2 modules for the miRNA input and additional 2 modules for the SMILES input as illustrated in the flowchart (Figure 5) as well as in the web server. Then, the input data will be pre-processed and used to predict the probabilities of SM-miRNAs regulation pairs.

### Validation of Prediction Models on Independent Datasets

Recently, a review summarized the developed small molecules to regulate miRNA expression (Van Meter et al., 2020). We selected the regulation pairs of small molecules on miRNA expression as the independent validation dataset. In the validation dataset, a total of 22 down-regulation pairs and 10 up-regulation pairs were included. Our down-regulation and up-regulation models successfully predicted 20 out of 22 down-regulation pairs (Supplementary Table S2) and 9 out of 10 up-regulation pairs (Supplementary Table S3), which suggested that the accuracy values of down-regulation and up-regulation models were 0.91 and 0.90 in the independent dataset.

### Selection of Oncogenic miRNAs and Molecular Docking

Based on the two cutoff values of |log2FC| > 1 and *p*-value < 0.05, a total of 195 up-regulated and 50 down-regulated miRNAs (Supplementary Figure S1A) were identified by exploiting the TCGA-UCEC dataset. The univariate Cox regression results suggested that 64 miRNAs (Supplementary Figure S1B) were negatively correlated with prognoses due to HR > 1 and *p*-value < 0.05. Based on these results, 11 miRNAs (Supplementary Figure S1B) were selected as oncogenic biomarkers since these RNAs were up-regulated in tumor samples and correlated negatively with prognosis.

**FIGURE 6:**
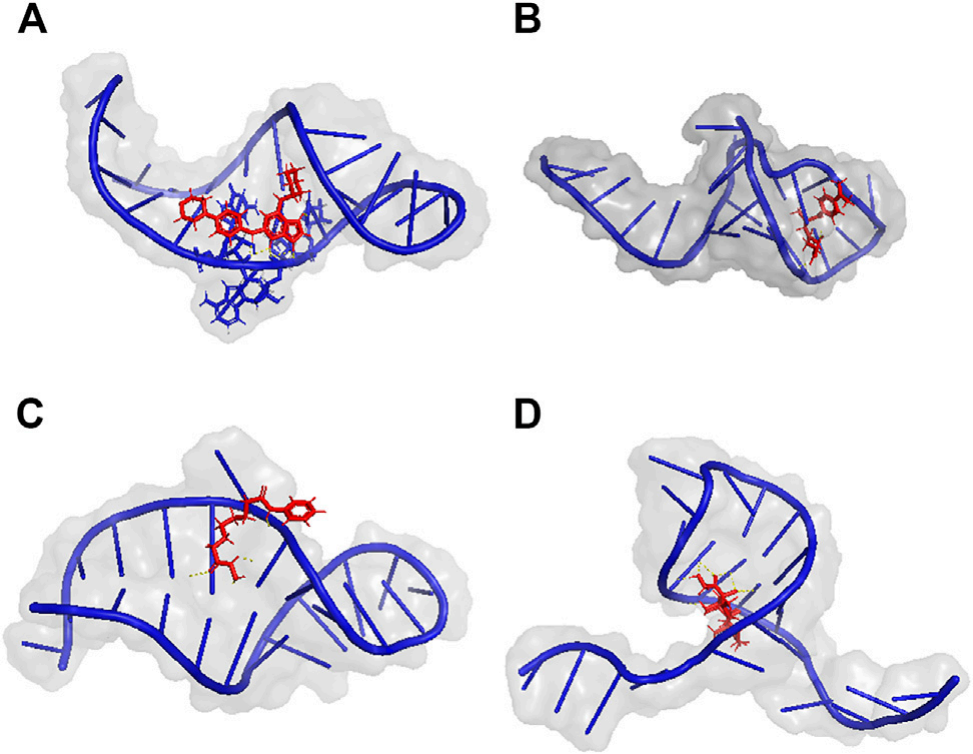
Molecular docking of CID210332 (hsa-miR-3131), CID444732 (hsa-miR-3614–3p), CID5311 (hsa-miR-3131), and CID5743 (hsa-miR-3616–3p). **(A)** An interaction between CID210332 and hsa-miR-3131 occurs at nucleotides 6–9. **(B)** An interaction between CID444732 and hsa-miR-3614–3p) occurs at nucleotides 5 and 12. **(C)** An interaction between CID5311 and hsa-miR-3131 occurs at nucleotides 5, 6, and 8. **(D)** An interaction between CID5743 and hsa-miR-3616–3p) occurs at nucleotides 8, 15, 16, and 17. The mall molecules, RNAs, and Hydrogen bonds are colored in red, blue, and yellow. Details about the lowest binding energy values are listed in Table 5.

Table 4 shows the top 3 predicted small molecules to regulate their oncogenic miRNAs using our model, as indicated by the highest rates. Among the 33 drug-miRNA regulation pairs, CID210332, CID444732, CID5311 and CID5743 were picked up for further studies because they were predicted to be able to down-regulate more than three miRNAs. According to the probabilities predicted using our model and available structure profiles from Pubchem, we classified the regulation pairs into two categories, with positive pairs including hsa-miR-3131 (CID210332), hsa-miR-3131 (CID5311), hsa-miR-3614–3p (CID444732), and hsa-miR-3616–3p (CID5743) and negative pairs including hsa-miR-3131 (CID6001), hsa-miR-3614–3p (CID6432), hsa-miR-3616–3p (CID13089). Based on molecular docking results obtained from AutoDock, we summarized the top 3 generated binding poses with the lowest free energy values for the selected drug-miRNA pairs (Table 5). Overall, the lowest binding energy values bore a striking resemblance to our prediction probabilities. For instance, the lowest binding energy values −7.12, −5.85, and −6.59 kcal/mol for has-miR-3131, has-miR-3614–3p, and has-miR-3616–3p (predicted positive pairs) strongly agreed to the quite high prediction probabilities of all above 0.8. By contrast, has-miR-3131, hsa-miR-3614–3p, hsa-miR-3616–3p (predicted negative pairs) gave the lowest binding energy values of −4.38, −1.16, and −4.49 kcal/mol in response to the small prediction probabilities. It has been established that a complex structure with lower binding energy is prone to be more stabilized than its counterparts that might be, even in many cases, disassembled. In combination, these results suggest that in terms of the binding profiles, the predicted positive pairs tighter than the negative pairs might be expected to be further exploited as more effective inhibitors or drugs. The binding poses of positive pairs with the lowest free energy values are shown in Figure 6 A-D.

**TABLE 4:** Top 3 small molecules are predicted to regulate their oncogenic miRNAs.

miRNA name	Sequence	Small molecule (rates)
hsa-miR-3074–5p	GUU​CCU​GCU​GAA​CUG​AGC​CAG	CID444732 (0.818)
		CID5311 (0.804)
		CID5743 (0.788)
hsa-miR-3131	UCG​AGG​ACU​GGU​GGA​AGG​GCC​UU	CID210332 (0.858)
		CID5311 (0.848)
		CID444732 (0.846)
hsa-miR-3170	CUG​GGG​UUC​UGA​GAC​AGA​CAG​U	CID8490 (0.89)
		CID3385 (0.842)
		CID210332 (0.82)
hsa-miR-3614–3p	UAG​CCU​UCA​GAU​CUU​GGU​GUU​UU	CID444732 (0.85)
		CID6036 (0.834)
		CID5793 (0.834)
hsa-miR-3614–5p	CCA​CUU​GGA​UCU​GAA​GGC​UGC​CC	CID5743 (0.896)
		CID60823 (0.876)
		CID60750 (0.87)
hsa-miR-3616–3p	CGA​GGG​CAU​UUC​AUG​AUG​CAG​GC	CID5743 (0.898)
		CID60750 (0.862)
		CID5790 (0.862
hsa-miR-3616–5p	AUG​AAG​UGC​ACU​CAU​GAU​AUG​U	CID5743 (0.782)
		CID3385 (0.76)
		CID5790 (0.754)
hsa-miR-449b-3p	CAG​CCA​CAA​CUA​CCC​UGC​CAC​U	CID210332 (0.76)
		CID60823 (0.76)
		CID5311 (0.754)
hsa-miR-449b-5p	AGG​CAG​UGU​AUU​GUU​AGC​UGG​C	CID6036 (0.802)
		CID5793 (0.802)
		CID5743 (0.784)
hsa-miR-4746–5p	CCG​GUC​CCA​GGA​GAA​CCU​GCA​GA	CID8490 (0.876)
		CID210332 (0.83)
		CID3385 (0.796)
hsa-miR-616–3p	AGU​CAU​UGG​AGG​GUU​UGA​GCA​G	CID444732 (0.814)
		CID2236 (0.78)
		CID5311 (0.778)

**TABLE 5:** Docking results of selected positive and negative pairs of miRNAs and small molecules.

miRNA	Drug	Binding poses	Binding energy (kcal/mol)	Prediction binding rate	Type
hsa-miR-3131	CID210332	1	−7.12	0.858	Positive
hsa-miR-3131	CID210332	2	−6.90	0.858	Positive
hsa-miR-3131	CID210332	3	−6.45	0.858	Positive
hsa-miR-3131	CID5311	1	−4.32	0.848	Positive
hsa-miR-3131	CID5311	2	−4.13	0.848	Positive
hsa-miR-3131	CID5311	3	−4.12	0.848	Positive
hsa-miR-3614–3p	CID444732	1	−5.85	0.85	Positive
hsa-miR-3614–3p	CID444732	2	−5.75	0.85	Positive
hsa-miR-3614–3p	CID444732	3	−5.75	0.85	Positive
hsa-miR-3616–3p	CID5743	1	−6.59	0.898	Positive
hsa-miR-3616–3p	CID5743	2	−5.93	0.898	Positive
hsa-miR-3616–3p	CID5743	3	−5.88	0.898	Positive
hsa-miR-3131	CID6001	1	−4.38	0.044	Negative
hsa-miR-3131	CID6001	2	−4.37	0.044	Negative
hsa-miR-3131	CID6001	3	−4.36	0.044	Negative
hsa-miR-3614–3p	CID6432	1	−1.16	0.044	Negative
hsa-miR-3614–3p	CID6432	2	−1.16	0.044	Negative
hsa-miR-3614–3p	CID6432	3	−1.14	0.044	Negative
hsa-miR-3616–3p	CID13089	1	−4.49	0.046	Negative
hsa-miR-3616–3p	CID13089	2	−4.07	0.046	Negative
hsa-miR-3616–3p	CID13089	3	−4.07	0.046	Negative

## Discussion

The human genome contains 20,000 to 25,000 protein-coding genes, but only 600 proteins could be drug targets for diseases (Hopkins and Groom, 2002). Most proteins could not be targeted or modulated by a drug molecule, and they are recognized as “undruggable” (Schmidt, 2014). However, intervention of miRNA related cellular processes could modulate these “undruggable” proteins *via* their miRNA gene regulators since 30% of proteins are indirectly regulated by miRNA (Schmidt, 2014). As a result, miRNAs are regarded as high-value drug targets. Currently, several strategies including antisense oligonucleotides (ASOs), miRNA sponges, CRISPR/Cas9, and small molecule inhibitors were adopted to modulate miRNAs (Wen et al., 2015). ASOs and miRNA sponge strategies failed because of poor delivery and decreased *in vivo* stability. CRISPR/Cas9 is associated with permanent harmful downstream effects and off-target effects (Wen et al., 2015). On the contrary, small molecules are more easily delivered, more stable, and have been successfully tested in clinical research and pharmacokinetic tests (Monroig et al., 2015). For example, small molecules represent around 75% of all drugs accepted by FDA.

There are two types of computational models that have been proposed to investigate the relations between small molecules and miRNAs. The first type of models used sequencing data to find the correlations between small molecules and miRNAs. For example, Small Molecule-MiRNA Network (SMirN) was constructed by combing links of genes-small molecule and links of genes-miRNAs. The links of genes-small molecule came from the results of differential expression of miRNA target genes by treatment with small molecules, and the links of genes-miRNAs came from miRNA-genes prediction tools (Jiang et al., 2012). Besides, another model named SmiRN-AD used mRNA-miRNA links and mRNA expression profiles (treated with small molecules) to provide the small molecule and miRNA association network in Alzheimer’s disease (Meng et al., 2014). Models of the second type were usually constructed by machine learning methods by utilizing the features of miRNAs and small molecules such as chemical structure similarities. For example, GISMMA, a graphlet interaction-based method for predicting small molecule and miRNA association, was proposed by leveraging the similarities of small molecules, the similarities of miRNAs, and known associations between small molecules and miRNAs (Guan et al., 2018). Another model “ELDMA” combined results of information, including small molecule chemical structure similarities, functional consistency, and miRNA target similarities. It used PCA and a convolutional neural network to extract features and construct the SVM classifier to infer new drug-miRNA relations (Deepthi and Jereesh, 2021).

Although the aforementioned models could predict the associations between small molecules and miRNAs, some major disadvantages exist in these models: 1) They could not predict the regulation of miRNA expression by small molecules (up-regulation or down-regulation). 2) An online web server based on the proposed model is not available, which limits the application of these models. 3) The prediction performance of models in the testing dataset is less satisfactory. For example, TLHNSMMA, a triple layer heterogeneous network to predict associations between miRNAs and small molecules obtained an AUC value of 0.81 on the testing dataset (Qu et al., 2018). In another study, a network-based framework SMiR-NBI achieved an AUC value of 0.82 by 10-fold cross validation (Li et al., 2016). 4) The number of small molecules and miRNAs that could be predicted by these models is limited. For example, only 831 SMs and 541 miRNAs were included in the TLHNSMMA work (Qu et al., 2018). and 5) Some models contain complex processes such as miRNA structure prediction (Disney et al., 2016). Thus, we implemented the user-friendly and accurate web server to meet the demand of predicting miRNAs that could be up-regulated/down-regulated by a certain small molecule, and of predicting small molecules that could target a certain miRNA.

In this study, we have developed a web server with random forest models that capture regulation relationships of small molecules with miRNAs expression, aiming to infer unknown small molecule−miRNA regulation associations. The advantages of our model and web server can be summarized as follows. 1) The experimental validated regulation pairs were split into miRNA up-regulation pairs and miRNA down-regulation pairs. Thus, the prediction models based on up-regulation pairs and miRNA down-regulation pairs could not only predict the association between a given small molecule and a miRNA, but also suggest whether the miRNA expression is increased or decreased by this small molecule. This prediction result is crucial for anti-tumor drug development since the oncogenic miRNAs should be inhibited and tumor suppressor miRNAs should be activated. 2) The web server provided in this study is freely available for any academic purposes of users. 3) Because of a comparatively rigorous scheme specifically designed for screening high-quality potential negative regulation pairs, the constructed random forest model demonstrated good performance on the testing (AUC of 0.911 for the up-regulation model and 0.896 for the down-regulation model) and validation datasets (the accuracy values of down-regulation and up-regulation models were 0.91 and 0.90). 4) More potential miRNAs and small molecules could be predicted by the web server. For example, we have provided a built-in cohort including a total of regulation pairs of 2,236 miRNAs and 1,509 small molecules. 5) Since our model is able to achieve a relatively high prediction performance by directly extracting features from miRNA sequences to then predict the probabilities of miRNAs regulated by small molecules, processes of leveraging structural features of miRNAs are not involved in our model development. Users only need to prepare miRNA sequences to predict regulation profiles by the small molecules, or alternatively prepare SMILES strings to predict regulation profiles by the miRNAs.

Uterine corpus cancer is a major cause of death worldwide, and 417,000 new cases and 97,000 deaths were recorded in 2020 (Sung et al., 2021). Uterine Corpus Endometrial Carcinoma (UCEC) represents 90% of uterine corpus cancer and the incidence of UCEC is increasing (Paleari et al., 2021). The main risk factors for EC are represented by the excess of exogenous and endogenous estrogens (Paleari et al., 2021). The selection of therapeutic strategy for UCEC mainly relies on clinical pathological risks such as the tumor stage. For early-stage UCEC patients, surgical treatment alone is recommended (Morice et al., 2016). Advanced-stage UCEC patients are recommended to be treated with chemotherapy after surgical intervention (Kupets and Le, 2013). However, the 5-years overall survival rate of stage IV UCEC patients is less than 30% (Morice et al., 2016). Therefore, novel targets and alternative therapeutic agents are urgently required for treating patients of advanced stage UCEC.

Based on miRNA sequencing data from the TCGA-UCEC dataset, 11 oncogenic miRNAs were selected. Using our web server, CID210332, CID444732, CID5311, and CID5743 were predicted to be able to decrease the expression of more than three miRNAs. CID210332 (Reversine), a 2,6-diamino-substituted purine analogue, has been reported to be effective in tumor suppression *via* induction of cell growth arrest and apoptosis of cancer cells (Park et al., 2019). CID444732 (Trichostatin A) was reported to effectively suppress the growth of endometrial cancer cells without toxic side effects (Takai et al., 2004). CID5311 (Vorinostat) is one of the histone deacetylase inhibitors and has antiproliferation and proapoptosis effects in endometrial cancer cells (Sarfstein et al., 2011). CID6743 (Dexamethasone) is utilized for treating inflammation and enhancing the antitumor efficacy of chemotherapeutic drugs. Dexamethasone is found to be a growth inhibitor for endometrial cancer cells (Davies et al., 2006). These results suggest that our web server could effectively provide anti-tumor drugs for endometrial cancer patients.

We acknowledge that there are some limitations of PSRR. Firstly, the number of positive/known regulation pairs is limited and might be on course to expand for constructing a model with better performance in the future. In this study, all known regulation pairs were downloaded from the SM2miR database. More regulation pairs from other resources could improve the prediction ability of the web server. Secondly, only the similarity scores between small molecules were taken into account on the course of generating the negative/unknown pairs. However, the similarity scores between miRNAs based on common targets and structures might contribute to generating higher-confidence negative/unknown pairs. The overall performance of PSRR illustrates that machine learning methods could boost the prediction capacities of modification of small molecules of miRNAs expression.

## Conclusion

Herein, we established PSRR, a powerful, user-friendly web server based on the random forest algorithm with the best accuracy for drug-miRNA regulation screening. The models in this web server demonstrated excellent predictive power in training, testing, case-study, and validation datasets. Furthermore, the server incorporates a database containing 1509 FDA-approved small molecules and 2,236 human miRNAs to provide more drug candidates. Procedures for prediction are simple and friendly, which makes it possible for users to only prepare miRNA sequences to predict the small molecules, or SMILES strings to predict the miRNAs. To our best knowledge, this is the first web server that could predict the effects on miRNA expression by small molecules. Overall, our study might contribute to developing novel potential therapeutic targets or treatments.

## Data Availability

The original contributions presented in the study are included in the article/Supplementary Material, further inquiries can be directed to the corresponding authors.

## Abbreviations

AGO, Argonaute; ANN, Artificial Neural Network; AUC, Area under the Curve; ASO, antisense oligonucleotidess; CV, cross-validation; GLM, Generalized Linear Models; HR, hazards ratio; KNN, K-nearest Neighbors; OS, Overall survival; RF, Random Forest; ROC, receiver operating characteristic; SMILES, simplified molecular-input line-entry system; SVM, Support Vector Machine; TCGA, The Cancer Genome Atlas; UCEC, Uterine Corpus Endometrial Carcinoma.
