# Supplementary material for: PSRR: A Web Server for Predicting the Regulation of miRNAs Expression by Small Molecules
Source: Front Mol Biosci. 2022 Mar 21;9:817294. doi: 10.3389/fmolb.2022.817294 (PMC8979021; doi:10.3389/fmolb.2022.817294)
Supplement: Supplementary file 1 [file DataSheet1.DOCX]

| Down-regulation Model | | Up-regulation Model | |
| --- | --- | --- | --- |
| threshold value | F1 score | threshold value | F1 score |
| 0.01 | 0.658733532 | 0.01 | 0.672674606 |
| 0.02 | 0.662376662 | 0.02 | 0.67595593 |
| 0.03 | 0.664939551 | 0.03 | 0.680143463 |
| 0.04 | 0.669274229 | 0.04 | 0.685996705 |
| 0.05 | 0.673371229 | 0.05 | 0.694694695 |
| 0.06 | 0.676963813 | 0.06 | 0.705802511 |
| 0.07 | 0.680567879 | 0.07 | 0.710184552 |
| 0.08 | 0.684539768 | 0.08 | 0.717504333 |
| 0.09 | 0.689468947 | 0.09 | 0.721368715 |
| 0.1 | 0.693877551 | 0.1 | 0.726312082 |
| 0.11 | 0.699908509 | 0.11 | 0.731465059 |
| 0.12 | 0.708333333 | 0.12 | 0.73676681 |
| 0.13 | 0.713685194 | 0.13 | 0.740634006 |
| 0.14 | 0.719132893 | 0.14 | 0.746453256 |
| 0.15 | 0.725023787 | 0.15 | 0.754231052 |
| 0.16 | 0.726315789 | 0.16 | 0.75805854 |
| 0.17 | 0.729898893 | 0.17 | 0.761656098 |
| 0.18 | 0.73220339 | 0.18 | 0.768186958 |
| 0.19 | 0.733560643 | 0.19 | 0.772968869 |
| 0.2 | 0.736070381 | 0.2 | 0.775697363 |
| 0.21 | 0.742237555 | 0.21 | 0.782675947 |
| 0.22 | 0.748508946 | 0.22 | 0.785547786 |
| 0.23 | 0.753259779 | 0.23 | 0.7881389 |
| 0.24 | 0.760506329 | 0.24 | 0.792125984 |
| 0.25 | 0.763358779 | 0.25 | 0.795409577 |
| 0.26 | 0.765848671 | 0.26 | 0.799681402 |
| 0.27 | 0.773291925 | 0.27 | 0.803377563 |
| 0.28 | 0.77486911 | 0.28 | 0.804198627 |
| 0.29 | 0.778012685 | 0.29 | 0.807973963 |
| 0.3 | 0.782515991 | 0.3 | 0.812781647 |
| 0.31 | 0.785368478 | 0.31 | 0.81383855 |
| 0.32 | 0.792165397 | 0.32 | 0.816936488 |
| 0.33 | 0.798245614 | 0.33 | 0.819055579 |
| 0.34 | 0.798007748 | 0.34 | 0.819823604 |
| 0.35 | 0.801339286 | 0.35 | 0.823080187 |
| 0.36 | 0.803370787 | 0.36 | 0.826552463 |
| 0.37 | 0.808607022 | 0.37 | 0.828953037 |
| 0.38 | 0.809142857 | 0.38 | 0.833913043 |
| 0.39 | 0.81571346 | 0.39 | 0.835309618 |
| 0.4 | 0.815635939 | 0.4 | 0.842198582 |
| 0.41 | 0.817699115 | 0.41 | 0.842901647 |
| 0.42 | 0.817204301 | 0.42 | 0.842671448 |
| 0.43 | 0.816596512 | 0.43 | 0.843199277 |
| 0.44 | 0.813067151 | 0.44 | 0.844929513 |
| 0.45 | 0.814678899 | 0.45 | 0.846012832 |
| 0.46 | 0.814814815 | 0.46 | 0.845227062 |
| 0.47 | 0.816070308 | 0.47 | 0.844673862 |
| 0.48 | 0.814720812 | 0.48 | 0.846880907 |
| 0.49 | 0.81202046 | 0.49 | 0.845605701 |
| 0.5 | 0.813384813 | 0.5 | 0.844868735 |
| 0.51 | 0.8125 | 0.51 | 0.843118383 |
| 0.52 | 0.808174028 | 0.52 | 0.841747573 |
| 0.53 | 0.811748999 | 0.53 | 0.84195122 |
| 0.54 | 0.807562458 | 0.54 | 0.840394089 |
| 0.55 | 0.799180328 | 0.55 | 0.838869608 |
| 0.56 | 0.792556857 | 0.56 | 0.83449651 |
| 0.57 | 0.79245283 | 0.57 | 0.834004024 |
| 0.58 | 0.785059901 | 0.58 | 0.830800405 |
| 0.59 | 0.782114975 | 0.59 | 0.827973456 |
| 0.6 | 0.780172414 | 0.6 | 0.825102881 |
| 0.61 | 0.768781911 | 0.61 | 0.821447163 |
| 0.62 | 0.765051395 | 0.62 | 0.812204103 |
| 0.63 | 0.76 | 0.63 | 0.80720339 |
| 0.64 | 0.753753754 | 0.64 | 0.80149413 |
| 0.65 | 0.75 | 0.65 | 0.799356223 |
| 0.66 | 0.740229885 | 0.66 | 0.791125541 |
| 0.67 | 0.725856698 | 0.67 | 0.779846659 |
| 0.68 | 0.718676123 | 0.68 | 0.774764804 |
| 0.69 | 0.70296237 | 0.69 | 0.764837626 |
| 0.7 | 0.688311688 | 0.7 | 0.759593679 |
| 0.71 | 0.667768595 | 0.71 | 0.749714286 |
| 0.72 | 0.646413502 | 0.72 | 0.737268519 |
| 0.73 | 0.618307427 | 0.73 | 0.722580645 |
| 0.74 | 0.598065084 | 0.74 | 0.710557533 |
| 0.75 | 0.578055308 | 0.75 | 0.7003003 |
| 0.76 | 0.549270073 | 0.76 | 0.691283293 |
| 0.77 | 0.522790698 | 0.77 | 0.675675676 |
| 0.78 | 0.496669838 | 0.78 | 0.657517155 |
| 0.79 | 0.478302797 | 0.79 | 0.636651871 |
| 0.8 | 0.454456415 | 0.8 | 0.619537275 |
| 0.81 | 0.42042042 | 0.81 | 0.596330275 |
| 0.82 | 0.38974359 | 0.82 | 0.570663094 |
| 0.83 | 0.365344468 | 0.83 | 0.557288136 |
| 0.84 | 0.336170213 | 0.84 | 0.531767956 |
| 0.85 | 0.312837109 | 0.85 | 0.505633803 |
| 0.86 | 0.285087719 | 0.86 | 0.474063401 |
| 0.87 | 0.258351893 | 0.87 | 0.441501104 |
| 0.88 | 0.230769231 | 0.88 | 0.412603151 |
| 0.89 | 0.206422018 | 0.89 | 0.381025249 |
| 0.9 | 0.184579439 | 0.9 | 0.340926944 |
| 0.91 | 0.158956109 | 0.91 | 0.299516908 |
| 0.92 | 0.141486811 | 0.92 | 0.259075908 |
| 0.93 | 0.114355231 | 0.93 | 0.221097046 |
| 0.94 | 0.088779285 | 0.94 | 0.159230096 |
| 0.95 | 0.0625 | 0.95 | 0.116696589 |
| 0.96 | 0.027989822 | 0.96 | 0.066481994 |
| 0.97 | 0.015364917 | 0.97 | 0.037523452 |
| 0.98 | 0.010269576 | 0.98 | 0.015180266 |
| 0.99 | 0.00257732 | 0.99 | 0.003816794 |

**Supplementary Table 1.** F1 scores of the down-regulation and up-regulation models based on different cut-off values.

| **miRNA** | **Sequence** | **Small Molecule** | **smile** | **Prediction rate** |
| --- | --- | --- | --- | --- |
| miR-21 | UAGCUUAUCAGACUGAUGUUGA | Diazobenzene compounds 1 | Nc2ccc(/N=N/c1ccc([N+](=O)O)cc1)cc2 | 0.638 |
| miR-21 | UAGCUUAUCAGACUGAUGUUGA | Diazobenzene compounds 2 | CCCNC(=O)c2ccc(/N=N/c1ccccc1)cc2 | 0.616 |
| miR-21 | UAGCUUAUCAGACUGAUGUUGA | streptomycin | C(N)N[C@@H]4[C@@H](O)[C@H](NC(=N)N)[C@H]3O[C@H]1*([C@H](C)[C@@](O)(C=O)[C@@H]1O[C@H]2C[C@@H](CO)[C@H](O)[C@H](O)[C@H]2NC)[C@@H]3[C@H]4O | 0.910 |
| Has-Let-7 | CUGUACAGCCUCCUAGCUUUCC | Compound 15 | CC4(C)Oc3cc(N2CCN(C(=O)OCc1ccccc1)CC2)c(N(=O)=O)cc3c5c4c*n5c6ccc(C(=O)O)cc6 | 0.542 |
| Has-Let-7 | CUGUACAGCCUCCUAGCUUUCC | KCB3602 | NC(=O)c3c(Nc1ccc(Cl)cc1Cl)[nH]c2c(Cl)ccc(Cl)c2c3=O | 0.582 |
| Has-Let-7 | CUGUACAGCCUCCUAGCUUUCC | 1632 | CC(=O)N(C)c3cccc(c2ccc1nnc(C)n1n2)c3 | 0.516 |
| miR-31 | AGGCAAGAUGCUGGCAUAGCU | analogues 17 | Cc2ccc(c1onc(C)c1C)cc2S(=O)(=O)N4CCc3ccccc34 | 0.58 |
| miR-31 | AGGCAAGAUGCUGGCAUAGCU | analogues 18 | Cc2ccc(c1onc(C)c1C)cc2S(=O)(O)n4cc(C)c3ccccc34 | 0.626 |
| miR-31 | AGGCAAGAUGCUGGCAUAGCU | analogues 19 | Cc2ccc(c1onc(C)c1C)cc2S(=O)(=O)N4CCc3ccccc3C4 | 0.64 |
| miR-373 | ACUCAAAAUGGGGGCGCUUUCC | PA-1 | CN(c1ccccc1)C3c2ccccc2N=C(CCCNCCCCNCCCN)C3(C)C | 0.384 |
| miR-25 | AGGCGGAGACUUGGGCAAUUG | isoliquiritigenin | O=C(/C=C/c1ccc(O)cc1)c2ccc(O)cc2O | 0.758 |
| miR-  301b | GCUCUGACGAGGUUGCACUACU | isoliquiritigenin | O=C(/C=C/c1ccc(O)cc1)c2ccc(O)cc2O | 0.568 |
| pre-miR-21 | UGUCGGGUAGCUUAUCAGACUGAUGUUGACUGUUGAAUCUCAUGGCAACACCAGUCGAUGGGCUGUCUGACA | ZINC00339240 | COc1ccccc1CC[NH2+]C3CC(=O)C(c2ccc(C)cc2)C3=O | 0.516 |
| pre-miR-21 | UGUCGGGUAGCUUAUCAGACUGAUGUUGACUGUUGAAUCUCAUGGCAACACCAGUCGAUGGGCUGUCUGACA | ZINC04574788 | NC(=O)/C/2=C/c1ccccc1CC2=[NH+]\CC(=S)Nc3ccccc3 | 0.516 |
| pre-miR-21 | UGUCGGGUAGCUUAUCAGACUGAUGUUGACUGUUGAAUCUCAUGGCAACACCAGUCGAUGGGCUGUCUGACA | ZINC05730110 | CC([NH3+])=C(C(C)=NNC(=O)c1ccccn1)C(=O)Nc2ccccc2 | 0.654 |
| pre-miR-21 | UGUCGGGUAGCUUAUCAGACUGAUGUUGACUGUUGAAUCUCAUGGCAACACCAGUCGAUGGGCUGUCUGACA | ZINC20030823 | O=c4cc([NH+]2CCC([NH+]1CCCC1)CC2)c3ccccc3o4 | 0.72 |
| pre-miR-21 | UGUCGGGUAGCUUAUCAGACUGAUGUUGACUGUUGAAUCUCAUGGCAACACCAGUCGAUGGGCUGUCUGACA | Doxorubicin | COc4cccc5c(=O)c3c(O)c2C[C@@](O)(C(=O)CO)C[C@H](O[C@H]1C[C@H](N)C(=O)[C@H](C)O1)c2c(O)c3c(=O)c45 | 0.806 |
| pre-miR-21 | UGUCGGGUAGCUUAUCAGACUGAUGUUGACUGUUGAAUCUCAUGGCAACACCAGUCGAUGGGCUGUCUGACA | Epirubicin | *N[C@H]5C[C@H](OC4CC(O)(C(O)CO)Cc3c(O)c2c(=O)c1cccc(OC)c1c(=O)c2c(O)c34)O[C@@H](C)[C@@H]5O | 0.814 |
| pre-miR-21 | UGUCGGGUAGCUUAUCAGACUGAUGUUGACUGUUGAAUCUCAUGGCAACACCAGUCGAUGGGCUGUCUGACA | Mitoxantrone | **7C(OC4C(O)[C@@H](*3(C1OC(CN)C(O)C(O)C1N)C2CC(N)CC(N)C23)O[C@@H]4CSCCN[C+2](C)(C)(=O)CC(C)(C)[NH2+]/C=C(N=N)/C(C)(C)OCCC(C)(CC)Oc6ccc5CC(=O)N(O)C(=O)c5c6)C(N)C7C(O)CCN | 0.644 |
| pre-miR-21 | UGUCGGGUAGCUUAUCAGACUGAUGUUGACUGUUGAAUCUCAUGGCAACACCAGUCGAUGGGCUGUCUGACA | AC1MMYR2 | N#CC1CNC(N)NC1N | 0.626 |
| pre-miR-21 | UGUCGGGUAGCUUAUCAGACUGAUGUUGACUGUUGAAUCUCAUGGCAACACCAGUCGAUGGGCUGUCUGACA | DIM | c4ccc3c(Cc1c[nH]c2ccccc12)c[nH]c3c4 | 0.394 |
| pre-miR-21 | UGUCGGGUAGCUUAUCAGACUGAUGUUGACUGUUGAAUCUCAUGGCAACACCAGUCGAUGGGCUGUCUGACA | Regorafenib | CNC(=O)c3cc(Oc2ccc(NC(=O)Nc1ccc(Cl)c(C(F)(F)F)c1)c(F)c2)ccn3 | 0.582 |

**Supplementary Table 2.** Results of predicting SM-miRNA down-regulation pairs. In the column “Prediction Result”, the SM-miRNA regulation pairs with a rate of higher than 0.41 are successfully predicted as binding or interacting.

| **miRNA** | **Sequence** | **Small Molecule** | **smile** | **Prediction rate** |
| --- | --- | --- | --- | --- |
| **hsa-mir-34a** | UGGCAGUGUCUUAGCUGGUUGU | Rubone | COc2cc(O)c(C(=O)/C=C/c1cc(OC)c(OC)cc1OC)c(OC)c2 | 0.638 |
| **hsa-miR-182-5p** | UUUGGCAAUGGUAGAACUCACACU | Kenpaullone | O=C4Cc1c([nH]c2ccc(Br)cc12)c3ccccc3N4 | 0.53 |
| **hsa-miR-182-5p** | UUUGGCAAUGGUAGAACUCACACU | Compound28 | NC4C=c3c2CC(=O)Nc1ccccc1c2[nH]c3=CC4 | 0.54 |
| **hsa-miR-1-5p** | ACAUACUUCUUUAUAUGCCCAU | Compound44 | O=C(c1ccccc1)c3c2ccccc2c5cccc4c(=O)ccc3c45 | 0.43 |
| **hsa-miR-15a-5p** | UAGCAGCACAUAAUGGUUUGUG | Compound45 | O=N(=O)c4ccc3nc(c1ccccc1)nc(c2ccccc2)c3c4 | 0.646 |
| **hsa-miR-15a-5p** | UAGCAGCACAUAAUGGUUUGUG | Compound46 | O=N(=O)c4ccc3nc(c1ccccc1O)nc(c2ccccc2)c3c4 | 0.782 |
| **hsa-miR-15a-5p** | UAGCAGCACAUAAUGGUUUGUG | Compound47 | O=N(=O)c4ccc3nc(c1ccccc1N(=O)=O)nc(c2ccccc2)c3c4 | 0.67 |
| **hsa-miR-192-5p** | CUGACCUAUGAAUUGACAGCC | Compound45 | O=N(=O)c4ccc3nc(c1ccccc1)nc(c2ccccc2)c3c4 | 0.574 |
| **hsa-miR-192-5p** | CUGACCUAUGAAUUGACAGCC | Compound46 | O=N(=O)c4ccc3nc(c1ccccc1O)nc(c2ccccc2)c3c4 | 0.62 |
| **hsa-miR-192-5p** | CUGACCUAUGAAUUGACAGCC | Compound47 | O=N(=O)c4ccc3nc(c1ccccc1N(=O)=O)nc(c2ccccc2)c3c4 | 0.598 |

**Supplementary Table 3.** Results of predicting SM-miRNA up-regulation pairs. In the column “Prediction Result”, the SM-miRNA regulation pairs with a rate of higher than 0.48 are successfully predicted as binding or interacting.
