# Supplementary figures and images for: PSRR: A Web Server for Predicting the Regulation of miRNAs Expression by Small Molecules
Source: Front Mol Biosci. 2022 Mar 21;9:817294. doi: 10.3389/fmolb.2022.817294 (PMC8979021; doi:10.3389/fmolb.2022.817294)

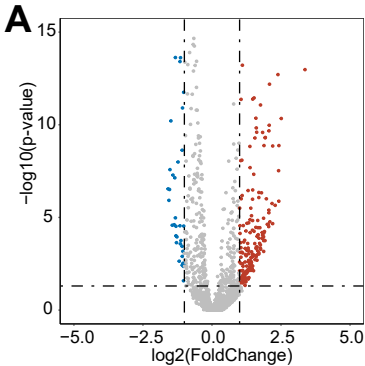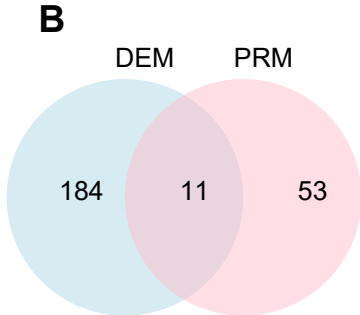

Supplement: Supplementary file 2 [file Image1.PDF]
